# A network-based approach for identifying suitable biomarkers for oral immunotherapy of food allergy

**DOI:** 10.1186/s12859-019-2802-9

**Published:** 2019-04-23

**Authors:** Jolanda H. M. van Bilsen, Lars Verschuren, Laura Wagenaar, Marlotte M. Vonk, Betty C. A. M. van Esch, Léon M. J. Knippels, Johan Garssen, Joost J. Smit, Raymond H. H. Pieters, Tim J. van den Broek

**Affiliations:** 1 TNO, PO Box 360, 3700 AJ Zeist, The Netherlands; 2 Institute of Risk Assessment Sciences, Utrecht University, Utrecht, The Netherlands; 3 Utrecht Institute of Pharmaceutical Sciences, Utrecht University, Utrecht, The Netherlands; 4 Danone Nutricia Research, Utrecht, The Netherlands

**Keywords:** Bayesian network analyses, Bioinformatics, Experimental food allergy, Oral immunotherapy, Topological data analyses

## Abstract

**Background:**

Oral immunotherapy (OIT) is a promising therapeutic approach to treat food allergic patients. However, concerns with regards to safety and long-term efficacy of OIT remain. There is a need to identify biomarkers that predict, monitor and/or evaluate the effects of OIT. Here we present a method to select candidate biomarkers for efficacy and safety assessment of OIT using the computational approaches Bayesian networks (BN) and Topological Data Analysis (TDA).

**Results:**

Data were used from fructo-oligosaccharide diet-supported OIT experiments performed in 3 independent cow’s milk allergy (CMA) and 2 independent peanut allergy (PNA) experiments in mice. Bioinformatical approaches were used to understand the data structure. The BN predicted the efficacy of OIT in the CMA with 86% and indicated a clear effect of scFOS/lcFOS on allergy parameters. For the PNA model, this BN (trained on CMA data) predicted an efficacy of OIT with 76% accuracy and shows similar effects of the allergen, treatment and diet as compared to the CMA model. The TDA identified clusters of biomarkers closely linked to biologically relevant clinical symptoms and also unrelated and redundant parameters within the network.

**Conclusions:**

Here we provide a promising application of computational approaches to a) compare mechanistic features of two different food allergies during OIT b) determine the biological relevance of candidate biomarkers c) generate new hypotheses to explain why CMA has a different disease pattern than PNA and d) select relevant biomarkers for future studies.

**Electronic supplementary material:**

The online version of this article (10.1186/s12859-019-2802-9) contains supplementary material, which is available to authorized users.

## Background

Food allergy is an important socio-economic and health problem estimated to occur in 6–8% of children and in 1–2% of adults [1–3]. Unfortunately, to date there is no effective and safe therapy available and only symptomatic treatment and elimination diets are currently available.

Human studies have shown that both subcutaneous immunotherapy (SCIT) and oral immunotherapy (OIT) which are based on the regular administration of the culprit food in increasing doses, have promising therapeutic potential in allergy. Even though SCIT may have some clinical efficacy for food allergy (increased food allergen thresholds), treatment has been shown to be associated with a high incidence of allergic side effects, which currently limits its application in clinical practice [4–6]. Few clinical trials have shown encouraging results of specific OIT in CM and PN allergic children [7–10]. OIT increases food allergen thresholds, diminishes skin prick test responses, enhances allergen-specific IgG4, decreases allergen-specific IgE, increases the activation threshold of basophils and temporarily increases regulatory T cells (Tregs) and relevant cytokine levels [7–9, 11]. OIT is considered safer than SCIT, and hence more suitable for human treatment [4, 8]. However, although OIT has some efficacy, it is hampered by the high incidence of allergic side-effects [7, 12–14]. Moreover, to date OIT has not yet resulted in long-lasting protection against food allergy: children subjected to OIT appear desensitized (i.e. protection against clinical effects), but not tolerized to peanut (i.e. induction of complete non-responsiveness or selective modulation of B and T cell responses) [9, 15], so the continuous ingestion of the allergen is still required to protect against clinical symptoms.

Data suggest that in addition to the food itself, an immune modulating agent (adjuvant) may be helpful to induce tolerance rather than desensitization [16–20]. In addition, an immune modulating agent may enhance the safety of the IT procedure by reducing the optimal allergen dose required to induce tolerance or by direct suppression of the allergic effector response over shorter treatment periods. Recent in vitro studies, as well as studies in animals and in allergic children, suggest that non-digestible carbohydrates, such as fructo-oligosaccharides (FOS) may improve both the efficacy and the safety of subcutaneous and oral therapeutic approaches. FOS has been shown to directly interact with the epithelium and to modulate the intestinal mucosal and systemic immune system from an allergic tuning towards a Treg and Th1 setting [21], thereby suppressing allergic inflammation [22–24].

One of the major challenges in immunotherapy of food allergy is the lack of food allergy-specific biomarkers for disease diagnosis, illness monitoring, therapy evaluation, and prognosis prediction. To improve our understanding and ability to intervene in complex multifactorial food allergy, it is important to investigate the molecular networks underlying the biological system and elucidate which interactions contribute to pathology and how this occurs. Biomarkers involved in these processes should be measurable indicators of normal biologic processes, pathogenic processes, or therapeutic responses, for the risk assessment, early diagnosis, and predicting and monitoring responses to therapies and toxicities.

This article focuses on applying data mining tools to search for hidden trends within large data sets. Here, Bayesian modeling in combination with technologies from topological data analysis and network science were used to analyze complex data from experimental OIT studies in mice by unraveling the complex relationships between analyzed parameters and prioritizing candidate biomarkers.

A Bayesian network (BN) is a type of probabilistic graphical model that lies at the intersection between statistics and machine learning. A BN is a compact representation of a probability distribution over a set of discrete variables. It can help to create a simplified overview of a complicated experiment, depicting an intuitive representation of relationships between variables, where it combines prior knowledge (such as the known relationships between variables) with data from observations. It captures the relationships between variables, and may be used to make inferences about unobserved variables. BNs are particularly suitable to deal with multiple cause-effect relationships within a complex system. Furthermore, a trained BN describing general relationships among variables can be used to make inferences about what-if scenarios and can as such be used to test hypotheses. Application of BNs has progressed enormously over the last decades leading to its use spanning all fields.

We use techniques that borrow extensively from topological data analysis and network science to extract information from high-dimensional data sets. The ‘shape’ of data, as it can be elucidated by topological analysis, can provide information about the observed system. The geometric shape of our application of these techniques corresponds to the way in which different features within the system interact. The use of Bayesian networks in combination with topological analysis enables the discovery of therapeutic mechanisms that trigger a specific cascade of processes underlying OIT and subsequently identify a wide range of relevant disease parameters. This way the study design of future studies may be optimized in silico, saving time and resources.

The aim of this study was i) to compare the key drivers of the mechanisms of scFOS/lcFOS diet-supported OIT in peanut allergy and cow’s milk allergy and ii) to identify the biological relevance of biomarker (panels) of immunotherapy of food allergy thereby enabling the prioritization of candidate biomarkers.

## Methods

### Data sources

**Fig. 1:**
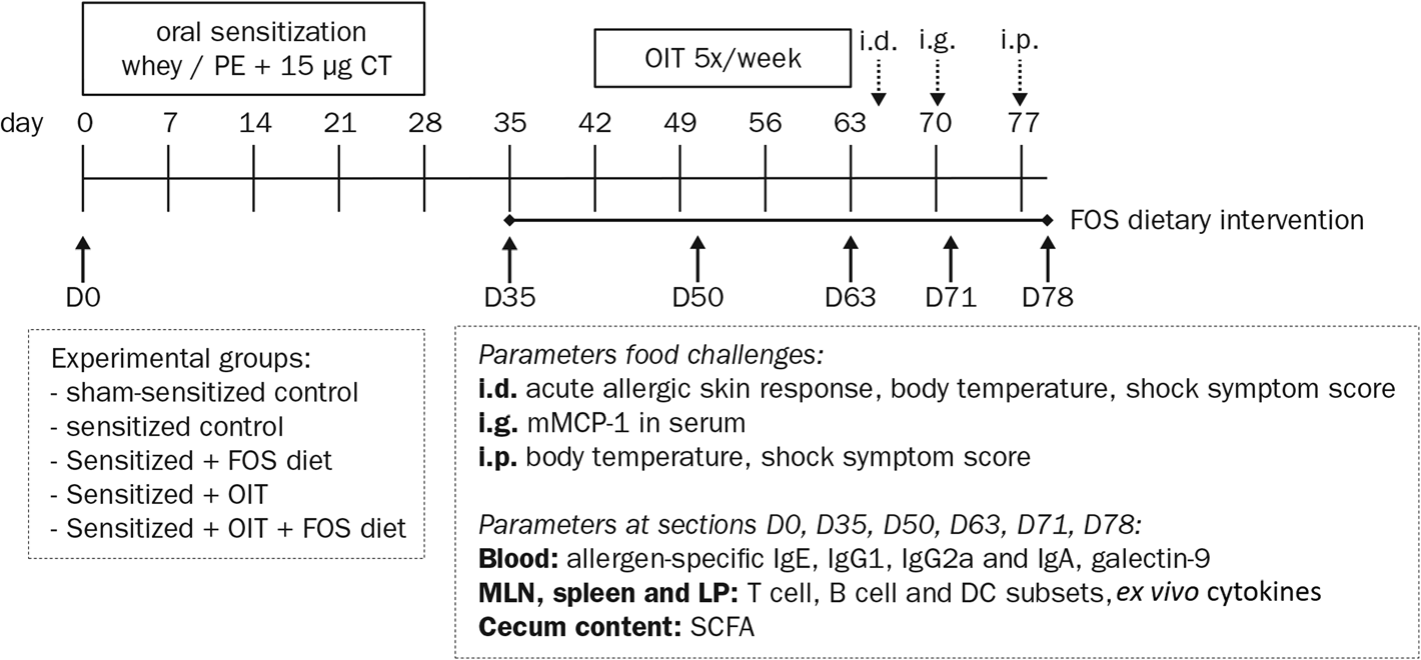
Experimental timelines of PNA and CMA models. 6-week-old female C3H/HeOuJ mice were randomly allocated to the control- and experimental groups: sham-sensitized control group;, sensitized control group;, FOS supplemented group; oral immunotherapy group; and the oral immunotherapy with FOS supplementation group. Mice were i.g. sensitized to the cow’s milk protein whey or PE (20 mg whey in 0.5 ml or 6 mg PE in 2 ml PBS) with cholera toxin as an adjuvant (15 μg in 0.5 ml PBS). The FOS supplemented diet was provided from D35 to the end of the protocol and OIT with 10 mg whey or 1.5 or 15 mg PE in 0.5 ml PBS was given from D42-D59 (5 oral gavages/week for 3 weeks). Acute allergic symptoms were measured upon i.d. challenge at D64 (10 μg whey or 1 μg PE in 20 μl PBS/ear), mast cell degranulation was measured upon i.g. challenge at D70 (50 mg whey or 15 mg PE in 0.5 ml PBS) and an i.p. challenge (50 μg whey or 100 μg PE in 200 μl PBS) was conducted at D77 to stimulate T cell responses prior to organ collection. At 6 time points throughout the animal experiment (D0, D35, D50, D63, D71 and D78), subgroups of mice from each control- and experimental group were killed by cervical dislocation and blood and organs were collected. PE; peanut extract, CT; cholera toxin, OIT; oral immunotherapy, FOS; fructo-oligosaccharides, i.d.; intradermal, i.g.; intragastric, i.p.; intraperitoneal, LP; lamina propria of small intestine, SCFA; short-chain fatty acids

Data were obtained from previously published studies describing experimental peanut allergy (PNA) and cow’s milk allergy (CMA) models, in which female C3H/HeOuJ mice were sensitized to the allergens and treated with/without OIT and fed a diet supplemented with/without f scFOS/lcFOS [23, 25]. The experimental procedures from these previously published murine studies were approved and conducted according to the guidelines determined by the Ethical Committee of Animal Research of Utrecht University (DEC2014.III.12.120 and AVD108002015212). The treatment efficacy was assessed with an intradermal (i.d.), intragastric (i.g.) and intraperitoneal (i.p.) food provocation. The outline of the studies is depicted in Fig. 1. The results of these murine studies indicated that scFOS/lcFOS supplementation improved the efficacy of OIT in cow’s milk allergic mice.

### Bayesian network analyses

For Bayesian data analysis, a selection of variables was made from both the CMA and PNA model datasets to ensure that the selected variables were present in all data and that these were measured under equivalent circumstances Additional file 1. Moreover, the selected variables were objective parameters that determine clinically relevant allergy symptoms (body temperature drop during anaphylaxis, ear swelling upon local challenge and mast cell activity (mMCP-1)) in combination with allergen-specific IgE and IgG1, both known to be upregulated in sensitized mice ([26, 27]). These clinically relevant variables were combined with specific IgE and IgG1, both known to be upregulated in sensitized mice [26, 27]. The model used here was trained using data from three CMA animal model datasets. In order to integrate the data to train a single model, some considerations had to be made. Because several variables, which were to be included, were measured in assays that use relative values, pooling the data for use in the model required normalization and discretization. In the three datasets, variables with relative measures were normalized by rescaling to a new range (min-max normalization), so that variables from different data sets are comparable. Variables with an absolute scale (such as temperature) were kept unchanged.

Because of the relatively small amount of data available for model training, it was decided to train a discrete Bayesian network. In a discrete Bayesian network, each node represents a variable. Each node contains a conditional probability table that represents the joint probabilities of the states of this node and the states of the parent nodes. In order to express the variables and their dependencies as conditional probabilities, the variables have to be discrete. Discretization was therefore performed, using ‘Hartemink’s algorithm’. This is a method of discretization that automatically finds quantiles that preserve and maximize mutual information among variables within the network. It was applied as implemented by package ‘bnlearn’ [28, 29].

The structure of the network was not taken from the data, but provided by expert knowledge. After the model structure was defined, conditional probabilities were estimated using maximum likelihood estimation as implemented in package ‘bnlearn’ [29]. Model performance was assessed using a multiclass area under the ROC curve algorithm by [30].

### Topological data analyses

Data were taken from the CMA and PNA model datasets for topological visualization. For the CMA model, two experiments were merged into one dataset while for the PNA model data from one experiment was used Additional file 1. During this merge, features were discarded when only one of the two datasets contained said feature. This was done to prevent situations where the similarity of two features cannot be determined because of mutually exclusive sample sets. The processing procedure for both datasets was identical.

Among the included variables were mucosal mast cell protease-1 (mMCP-1) upon i.g. food challenge and allergen-specific IgE, IgG1, IgG2a in serum, acute allergic skin response (ear swelling) upon i.d. food challenge, anaphylaxis symptom score and body temperature after challenge, leukocyte phenotypes by flowcytometry, cytokine release of MLN, LP and spleen-derived lymphocytes after ex vivo stimulation with anti-CD3, whey protein or peanut extract (PE) and short-chain fatty acids (SCFA).

To construct the graph, an adjacency matrix was calculated using Spearman’s rank correlation similarity. Using the resulting adjacency matrix, a mutual k-nearest neighbors graph was constructed (as described by [31]). The same publication shows that the graph, given large enough n, will be connected if we choose k on the order of log(n) where n is the number of samples in the data. Therefore, for each of the datasets, k equaled log(n).

To aid visualization and interpretation of the mutual nearest neighbors network, the nodes of the network were assigned to clusters using the multilevel modularity optimization algorithm by [32].

## Results

### Bayesian network illustrates beneficial effects of scFOS/lcFOS diet and OIT on CMA

Internal validation of the performance of the BN-model trained on the CMA model data was performed to assess how well the model can infer the clinical severity of allergy in light of the diet and treatment effects. mMCP-1 was chosen as a meaningful objective representation of allergic severity. The predictive performance of the model on mMCP-1 tertiles from CMA model data was good, as assessed from a multiclass area under the ROC curve value of 0.86. The ROC curves from the predicted mMCP-1 tertiles of the CMA data are depicted in Additional file 2).

**Fig. 2:**
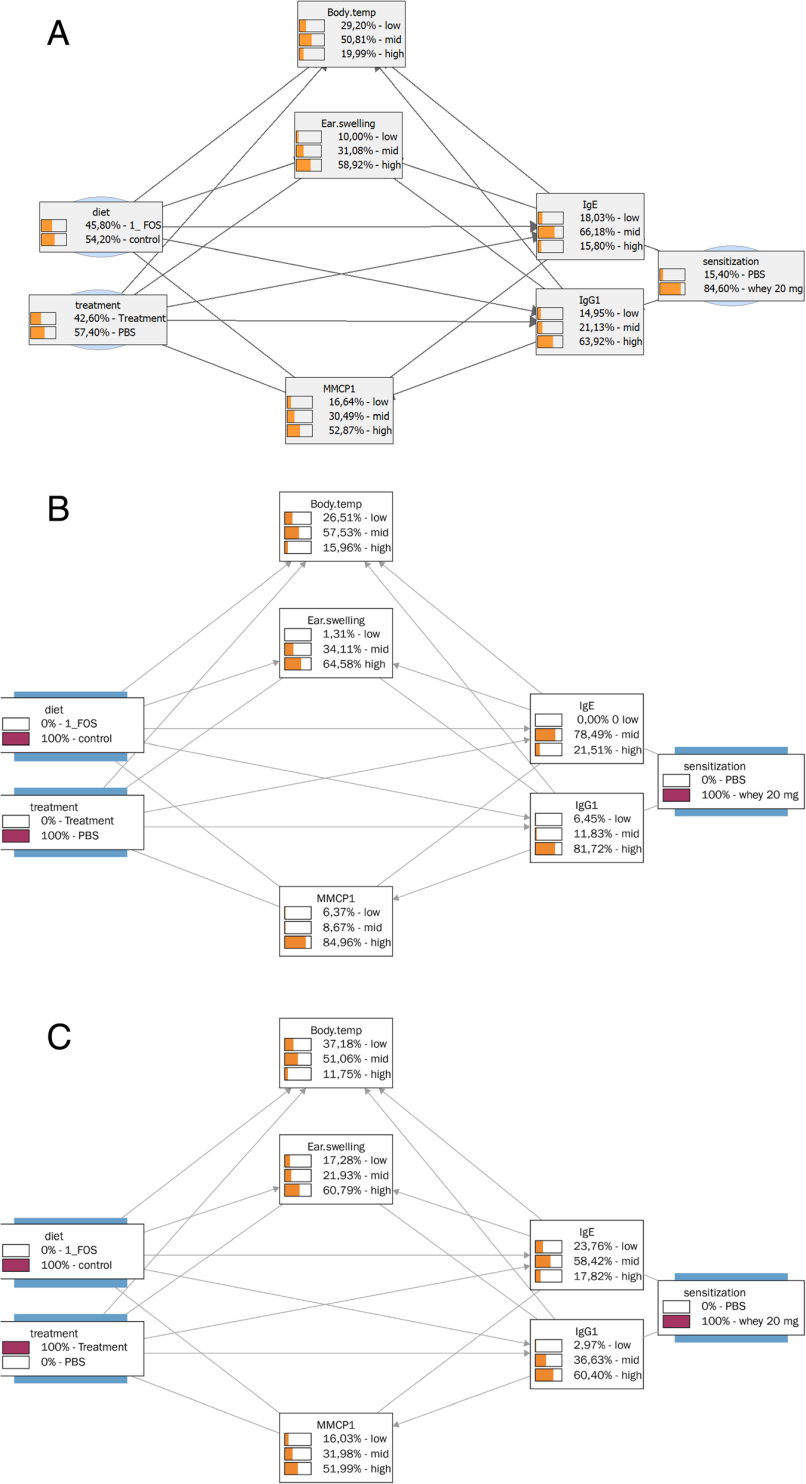
Bayesian network trained on CMA data, effects of sensitization and/or OIT. BN depicting the relationships between the analyzed parameters and the animal treatments (sensitization, scFOFS/lcFOS diet, or OIT treatment) using CMA data. Moreover the probability distributions of all BN variables are depicted **a**) irrespective of animal treatments, **b**) assuming that all animals were sensitized and **c**) assuming that all animals were sensitized and received OIT

Next we used the BN to make inferences to test the influence of sensitization with or without OIT on the probability distribution of BN variables. In Fig. 2a the probability distribution of the BN parameters is shown irrespective of the animal treatments (sensitization, scFOS/lcFOS dietary supplementation, or OIT), showing 84.6% probability of being sensitized, 54.2% probability of having received the scFOS/lcFOS supplemented diet and 42.6% probability of having received OIT treatment and the other probability distributions of the parameters analyzed in the animals.

The probability distributions of the analyzed parameters changed upon the assumption of the model that all animals were sensitized (Fig. 2b) resulting in only a small shift in probability distributions of the variables. This can be explained by the fact that the chance of sensitization irrespective of the animal treatments was already 84.6% (Fig. 2a), so the increase to 100% sensitization does not have a major effect. The effect on the probability distributions change significantly upon the assumption of the model that all animals were sensitized and received OIT treatment (Fig. 2b and c). This results in a clear decrease in probability of high specific-IgE (from 21.51 to 17.82%), specific-IgG1 (from 81.72 to 60.40%), ear swelling upon i.d. challenge (from 64.58 to 60.79%) and mMCP-1 (from 84.96 to 51.99%) levels, indicating the clear effect of OIT on these allergy parameters.

**Fig. 3:**
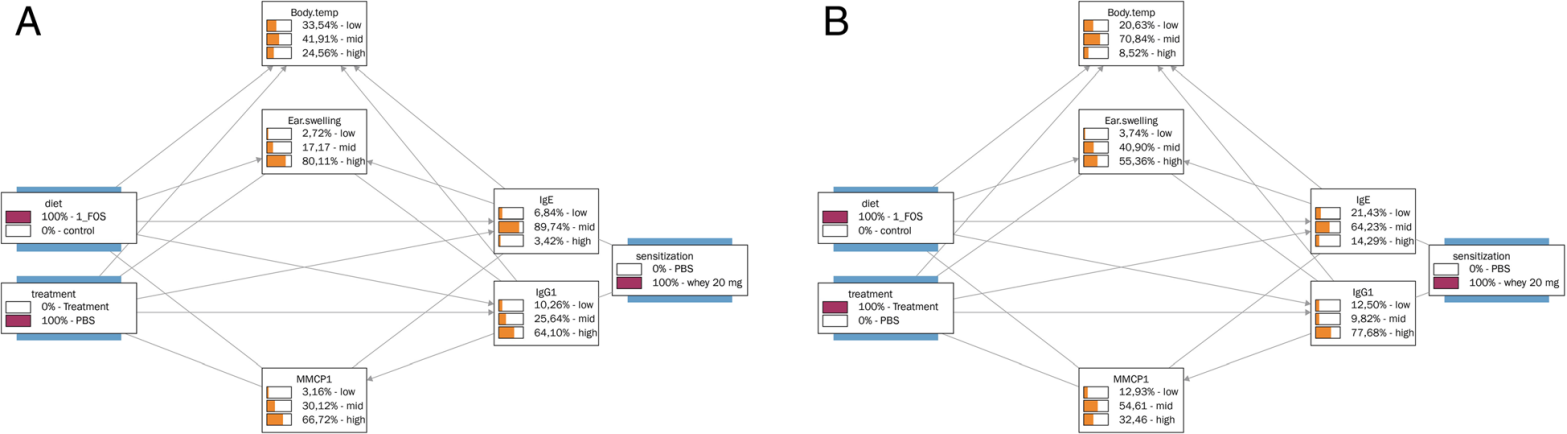
Bayesian network trained on CMA data, effects of scFOS/lcFOS diet with or without OIT. BN depicting the relationships between the analyzed parameters and the animal treatments (sensitization, scFOFS/lcFOS diet, OIT treatment) using the CMA data. The probability distributions of all BN variables are depicted assuming that all animals were sensitized and **a**) received scFOS/lcFOS diet or **b**) received scFOS/lcFOS diet in combination with OIT

Next we investigated the effect of the scFOS/lcFOS supplemented diet on the probability distributions in sensitized animals (Figs. 2b and 3a). This modeling showed a clear decrease in probability of high specific-IgE (from 21.51 to 3.42%), high specific-IgG1 (from 81.72 to 64.10%), high mMCP-1 (from 84.96 to 66.72%) and an unexpected increase in high ear swelling (from 64.58 to 80.11%). Together, these calculations indicate a clear effect of scFOS/lcFOS diet on these allergy parameters. This effect of the scFOS/lcFOS diet on the probability distributions in sensitized animals is further increased by the extra addition of OIT in the model (Fig. 3a and b). %). It increased high specific IgE (3.42 to 14.29%) and IgG1 (64.10 to 77.68%) titers, whereas it also increased the beneficial low specific IgE (6.84% to 21.43), illustrating the mixed effect of adding OIT to the scFOS/lcFOS on IgE titers., however IgE-titers reflect sensitization and not the allergic status as illustrated by the fact that non-allergic individuals may have high IgE titers. Moreover, desensitized humans often have high IgE titers, but are no longer allergic. Therefore the relevance of IgE titers to monitor OIT efficacy is quite speculative [33]. Besides changes in IgE-titer, the addition of OIT to scFOS/lcFOS diet also results in a beneficial decreases in probability of high earswelling (from 80.11% to 55.36), high mMCP-1 (66.72 to 32.46%) and a decrease in anaphylaxis-associated drop in body-temperature (from 33.54% to 20.63).

Together these data indicate clearly the added effect of supplementing the diet with scFOS/lcFOS on the efficacy of OIT in CMA, which confirms previous work [23]. These examples illustrate how the BN is suitable to easily test and generate hypotheses by visualizing the consequences of what-if scenarios. Instead of analyzing the effects of interventions on the entire population, the modeling also enables to focus on the effects of interventions on subpopulations of subjects, which clearly makes this approach even more valuable for future applications (e.g. mechanism elucidation, patient stratification), although this goes beyond the scope of this manuscript.

### Bayesian network indicates similar key drivers in PNA and CMA

Although both CMA and PNA seem clinically similar diseases, they differ in the fact that CMA is most prevalent during early childhood, but is often outgrown [34] while PNA is more persistent and is the most frequent cause of life-threatening allergic reactions in adults [35].

To assess whether key features of the CMA and PNA models have similar properties, we used the Bayesian network that was trained on data from the CMA model to make inferences on the PNA model data. Inferences on mMCP-1 tertiles in the BN from a PNA model experiment, using the network trained on the CMA model data give a multiclass area under the ROC curve value of 0.76; which is considered a fair performance. The ROC curves from the predicted mMCP-1 tertiles of the PNA data are depicted in Additional file 3).

From these analyses, it is reasonable to deduce that the effects of the allergen, treatment and diet are similar within our Bayesian network abstraction of CMA across peanut and cow’s milk models. Therefore, we can regard the model as a reasonable representation of the core mechanisms and characteristics of allergy, treatment and diet.

### Topological data analyses prioritize candidate biomarkers

The BN indicated that the core characteristics of allergy, treatment and diet in both the PNA and CMA models are similar. Because many more parameters were analyzed throughout the course of each study in each individual mouse, the next question was whether it would be possible to indicate whether the relevance of the analyzed parameters differ between PNA and CMA by determining how the parameters are related to each other and to the clinical endpoints of the food allergy.

**Fig. 4:**
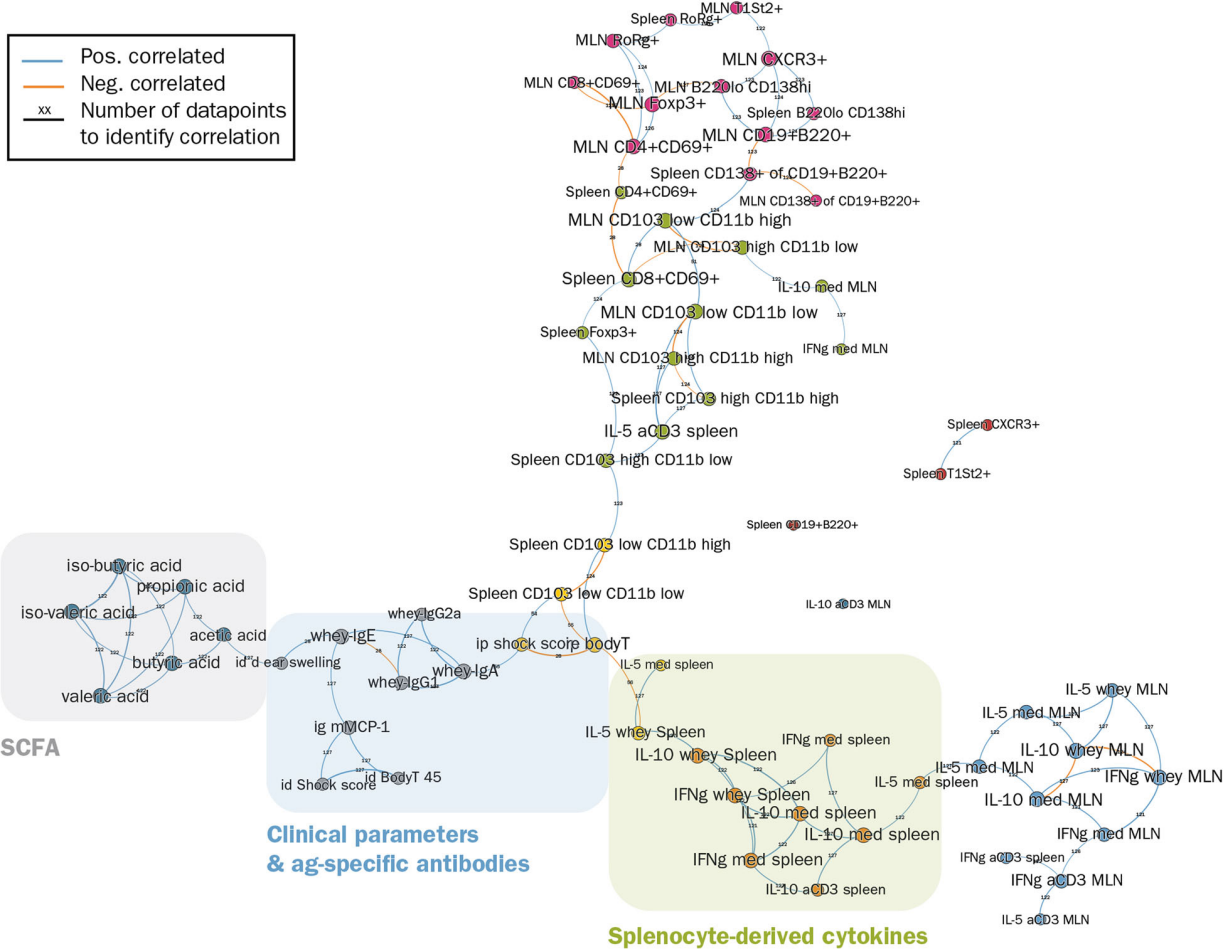
The mutual nearest neighbors network of the CMA model. Topological network showing the clustering of parameters (dots with same color). The clusters were used to identify the cluster-relationships in CMA. Moreover, the encircled clusters were used to compare the cluster-relationships between CMA and PNA (see Fig. 5)

Figure 4 shows the overview of the mutual nearest neighbors network of parameters that were measured in the CMA model. From this experiment, 66 parameters were available for analysis. These were organized in 7 large (6–12 parameters) and 4 small clusters (1–2 parameters). The parameters closest related to clinical outcomes (ear swelling, i.d. shock scores, i.d. body temperature, mMCP-1), were located in the same cluster (grey) indicating how closely they are connected to the antigen-specific antibodies. The remaining parameters closely linked to clinical outcomes (shock score and body temperature after i.p. challenge) were clustered together with splenocyte-derived parameters, both located in a cluster (yellow) which was quite closely linked to this antibody/clinical parameter-cluster.

The antibody/clinical parameter-cluster (grey) was closest related to SCFA-cluster (cadetblue) and to a cluster with spleen-derived/clinical parameters (yellow). The remaining clusters harboring MLN and splenocyte-derived parameters showed quite indirect relationships clusters (blue, orange, magenta, green,) with the antibody/clinical parameter cluster or had no relationship (dark blue, brown, indianred) at all (IL10 production of anti-CD3 stimulated MLN-derived lymphocytes, CD19 + B220+ cells, spleen CXCR3+ cells and spleen T1ST2+ cells). The indirect or absent relationship with clusters harboring clinical parameters indicates that they are of lesser importance to the process of OIT of food allergy.

**Fig. 5:**
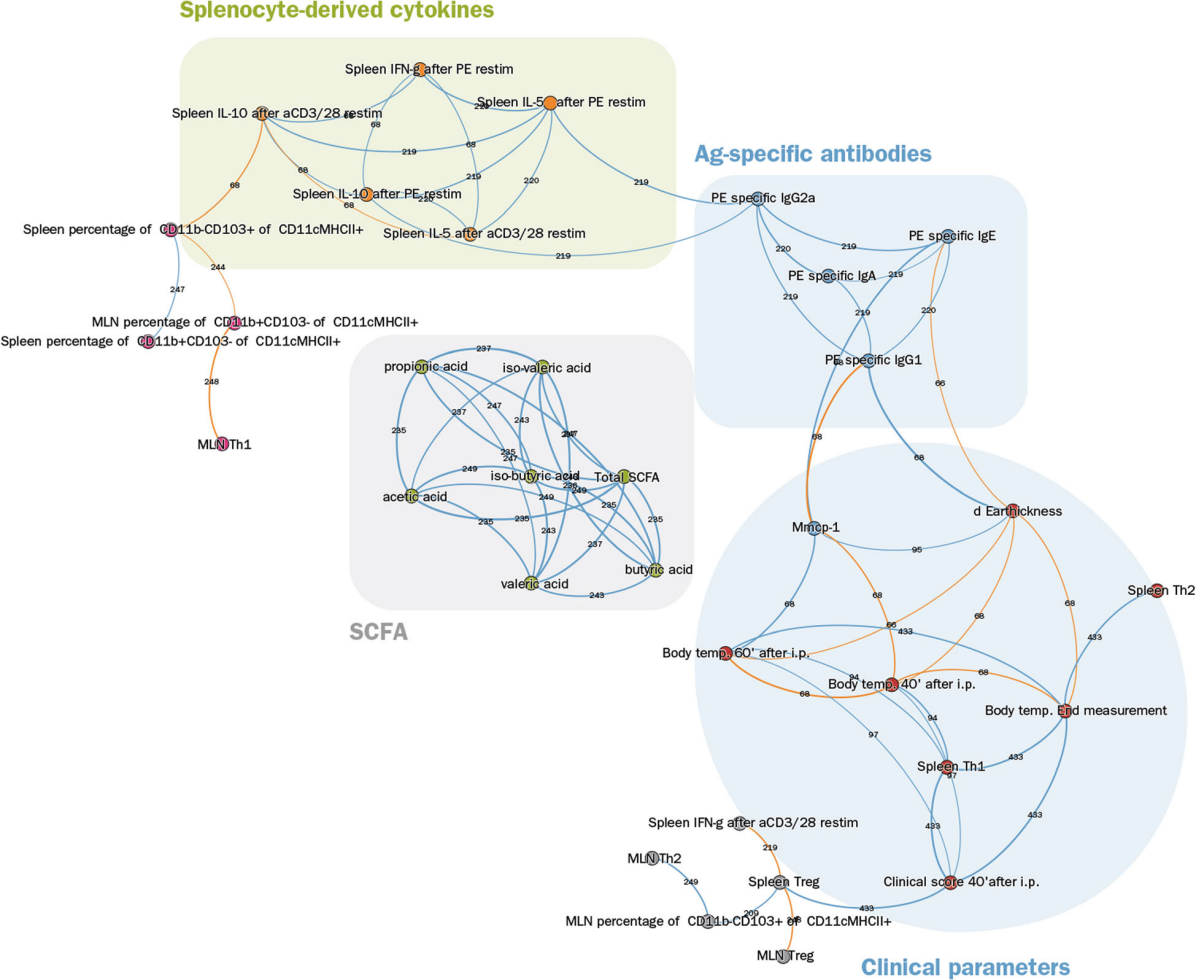
The mutual nearest neighbors network of the PNA model. Depicted is the topological network showing the clustering of parameters (dots with same color). The clusters were used to identify the cluster-relationships in CMA. Moreover, the encircled clusters were used to compare the cluster-relationships between PNA and CMA (see Fig. 4)

Figure 5 shows the TDA of the PNA model. In the PNA model 33 parameters were analyzed which were organized in 6 clusters containing 4–7 parameters. The parameters most closely related to clinical outcomes (ear swelling, body temperature, shock scores, mMCP-1) were located in the same cluster as spleen Th1 (CD183 + CD69+) and Th2 (T1ST2+/CD69+) cells. In contrast to the CMA model, all clinical parameters were located in the same cluster (indianred). Moreover, the antigen-specific antibodies were not linked in the same cluster as the clinical parameters, although they were closely linked (blue cluster). Another cluster (grey) closely linked to the clinical parameter cluster, consisted of the Tregs of spleens and MLN, Th2 cells in MLN, IFNγ production of antiCD3/CD28 stimulated spleens and the % CD11b-CD103+ cells of CD11c + MHCII+ DCs in MLN. The cluster (orange) harboring parameters of cytokine-production of stimulated splenocytes was indirectly linked to the clinical symptoms via the antibody cluster (blue). The remaining clusters showed quite an indirect relationship (magenta) with the clinical parameter cluster or had no relationship at all (green: SCFA-cluster). The latter finding is quite remarkable, since in the CMA model the SCFA-cluster was quite closely linked to the clinical parameter clusters.

In summary, even though not all analyzed parameters were identical in both models, there is a substantial similarity in topology and clustering of features from both PNA and CMA models (clinical parameters, antibodies, SCFA, cytokines from stimulated splenocytes) and the connections between the clusters, indicating that both models have largely similar mechanistic relationships. Nevertheless, topological data analyses also indicate differences between parameters and the clinical outcome (e.g. importance of SCFA). These differences identified by the mutual nearest neighbors networks may be useful to generate new hypotheses for observed clinical differences and prognoses of CMA and PNA.

## Discussion

Immunotherapy is currently the most promising therapy for patients with food allergy, who now rely on avoidance and carrying adrenaline auto injectors in case of accidental exposure. Unfortunately, current immunotherapy treatments of food allergy are too often accompanied by allergic side effects and do not appear to give long-term protection (reviewed by [17]). Recent in vitro studies, studies in animal models and studies in children with atopic dermatitis indicate that the addition of non-digestible sugars may improve the efficacy and safety of therapeutic approaches [22, 24, 36, 37]. However, the mechanism of action of these approaches is still largely unclear and as a result possibilities, limitations and safety / risks of these types of interventions are not known. The lack of insight into the mechanism also results in a large number of parameters being measured in studies, while the usefulness of results for a large part of these parameters is unclear and the studies become extremely elaborate.

Here we use ways of data mining to search for hidden trends within existing sets of data, by applying computational solutions (including algorithms, models and tools) which can be used to optimize experimental designs, data analyses and interpretation and hypotheses generation. We show that network analysis methods can be applied to investigate the underlying molecular mechanisms involved in immunotherapy of food allergy and the prioritization of biomarkers. By applying Bayesian networks and topological data analyses, ‘hidden’ information was discovered in the available data by visualizing the complex relationships between measured parameters and symptoms. In this study, we analyzed data animal experiments with the major allergenic foods, peanut and cow’s milk, which show different disease patterns. CMA is most prevalent during early childhood, but is often outgrown [34] while PNA is more persistent and is the most frequent cause of life-threatening allergic reactions in adults [35]. Our analyses suggest that the mechanisms involved in immunotherapy of CMA and PNA are very similar but not completely identical on the basis of the measured parameters. Possibly, slight differences can help to explain differences between patients.

One of the most striking differences was that our data clearly indicates the role of SCFA in CMA, but not in PNA. Previously, we have shown that in PNA and CMA, increased levels of SCFA, specifically butyrate, coincided with allergy reduction [23, 25]. These findings are confirmed in literature, where accumulating evidence indicates that SCFA have several anti-allergic properties by among others Treg induction and enhancement of the gut barrier function (as reviewed by [38]). Previous findings also show that dietary fibers which are metabolized by the gut microbiota into SCFA are able to downregulate PNA [39] and inflammatory airway responses in asthma [40]. Moreover, in CMA, levels of fecal butyrate were increased in tolerant infants [41]. So even though we have observed that increased levels of SCFA coincided with an allergy reduction in both PNA and CMA using the dataset used in current analyses ([23, 25], we here show that the structure of how the experimental data are correlated with each other are different between the allergy models. This means that the relationship between the SCFA and the clinical outcomes in PNA a) is more indirect and/or b) occurs via different mechanisms or parameters which were not analyzed in the studies and/or c) is not essential for the outcome of the allergy, so the level of SCFA could be an epiphenomenon which is in contrast to the current opinion in literature as mentioned before. This example nicely illustrates how these types of network-based analyses, enable the generation of new hypotheses, in this case the role of the different biomarkers in (treatment of) food allergy and to explain the differences between the disease patterns of CMA and PNA.

Another important feature of applying these types of network analyses is that they create a new view of the dataset which can be used to determine the biological relevance of the measured parameters. Using the mutual nearest neighbors networks from the topological data analyses, several criteria can be applied to prioritize the measured parameters: i) it became clear that several more or less ‘standard’ study parameters seem to have little relevance because they had no clear link to the clinical outcomes of immunotherapy, while others had a very direct link; ii) clusters of parameters were identified that individually were linked in a comparable manner to the biologically relevant parameters, so one could argue that analyses of only a few cluster-members would be sufficient instead of analyzing the entire panel; iii) mutual nearest neighbors networks enabled the prioritization of parameters based on the invasiveness of the measurements of the parameter in case of ‘equally’ relevant linked parameters to the clinical parameters. For instance, SCFA analyses in cecum content or IgE in serum are far less invasive for the subject than determining the skin response upon challenge, both in experimental animal models and in humans.

The application of the computational approaches demonstrated here allows investigators to more productively mine the currently-available and/or future data sets of phenotypes for food allergy-related traits to discover testable hypotheses for physiological mechanisms that lead to a food allergic phenotype. Other network-based methodologies to mine data to search for hidden trends within large data sets have been successfully applied in different fields. Most prominently in cancer research: recently, a cancer hallmark network framework for modeling genome sequencing data to predict cancer clonal evolution and associated clinical phenotypes has been generated and applied [42, 43], clearly indicating the high potency of network approaches to truly help further understanding of the complex nature of biological processes and translating the information into clinical practice.

Here we show that the addition of oligosaccharides with or without immunotherapy reduced the food allergy in both CMA and PNA. Moreover, even though the analyzed parameters in CMA and PNA were not identical, we showed that the key mechanisms between CMA and PNA are comparable. The BN shown here is quite simple in this experimental setting containing a limited set of parameters. For future clinical applications, it would be very interesting to expand this BN with patient characteristics (e.g. epigenetic factors, genetic factors, age, sex, medication), analyzing multiple parameters on multiple time points. This would result in a so called dynamic BN which would enable a stratification strategy to predict before the start of treatment whether a patient will benefit from undergoing immunotherapy. These new insights provide good starting points for selecting relevant biomarkers to monitor and predict safety and efficacy in later clinical studies, but also eventually in clinical applications.

## Conclusions

Here we provide a promising application of bioinformatics method to compare mechanistic features between different food allergies and to identify the biological relevance of biomarker (panels) of immunotherapy of food allergy. We have shown that the key drivers that influence PNA and CMA are similar but that these phenotypically similar diseases show mechanistic differences in their subnetworks. The application of this method may be useful to generate new hypotheses to explain why CMA has a different disease pattern than PNA and to select biomarkers that are useful in for future clinical studies.

## Additional files

Additional file 1: List of analyzed features of the CMA-model and PNA-model, including the number of animals per experiment and the analyzed parameters per model. (XLSX 15 kb)

Additional file 2: Receiver operating characteristic (ROC) curves for the predicted mMCP-1 tertiles of the CMA data. Data were obtained from 91 animals. The curves correspond to a multiclass area under the curve value of 0.86, as calculated using the algorithm in [30]. (TIFF 365 kb)

Additional file 3: Receiver operating characteristic (ROC) curves for the predicted mMCP-1 tertiles of the PNA data. Data were obtained from 67 animals. The curves correspond to a multiclass area under the curve value of 0.76, as calculated using the algorithm in [30]. (TIFF 370 kb)

## Abbreviations

Ag: Antigen
AIT: Antigen-specific immunotherapy
BN: Bayesian network
CMA: Cow’s milk allergy
CT: Cholera toxin
FOS: Fructo-oligosaccharides
FoxP3: Forkhead box protein 3
i.d.: intradermal
i.g.: intragastric
i.p.: intraperitoneal
LP: Lamina propria
MLN: Mesenteric lymph nodes
mMCP-1: Murine mast cell protease-1
OIT: Oral immunotherapy
PE: Peanut extract
PNA: Peanut allergy
SCFA: Short-chain fatty acid
scFOS/lcFOS: short-chain fructo-oligosaccharides / long-chain fructo-oligosaccharides
TDA: Topological data analyses
Th: T helper cell
Treg: Regulatory T cell

## References

[1] Carrard A, Rizzuti D, Sokollik C (2015). Update on food allergy. Allergy Eur J Allergy Clin Immunol.

[2] Prescott SL, Pawankar R, Allen KJ, Campbell DE, Sinn JKH, Fiocchi A (2013). A global survey of changing patterns of food allergy burden in children. World Allergy Organ J.

[3] Sampson HA (2004). Update on food allergy. J Allergy Clin Immunol.

[4] Nelson HS, Lahr J, Rule R, Bock A, Leung D (1997). Treatment of anaphylactic sensitivity to peanuts by immunotherapy with injections of aqueous peanut extract. J Allergy Clin Immunol.

[5] Oppenheimer JJ, Nelson HS, Bock SA, Christensen F, Leung DYM (1992). Treatment of peanut allergy with rush immunotherapy. J Allergy Clin Immunol.

[6] Chiang D, Berin MC (2015). An examination of clinical and immunologic outcomes in food allergen immunotherapy by route of administration. Curr Allergy Asthma Rep.

[7] Nowak-Wegrzyn A, Fiocchi A (2010). Is oral immunotherapy the cure for food allergies?. Curr Opin Allergy Clin Immunol.

[8] MacDonald TT, Di Sabatino A (2009). The immunologic basis for gastrointestinal food allergy. Curr Opin Gastroenterol.

[9] Jones SM, Pons L, Roberts JL, Scurlock AM, Perry TT, Kulis M (2009). Clinical efficacy and immune regulation with peanut oral immunotherapy. J Allergy Clin Immunol.

[10] Blumchen K, Ulbricht H, Staden U, Dobberstein K, Beschorner J, de Oliveira LCL (2010). Oral peanut immunotherapy in children with peanut anaphylaxis. J Allergy Clin Immunol.

[11] Larenas-Linnemann DD (2009). Certainties and doubts about sublingual and oral immunotherapy in children. Curr Opin Allergy Clin Immunol.

[12] Thyagarajan A, Varshney P, Jones SM, Sicherer S, Wood R, Vickery BP (2010). Peanut oral immunotherapy is not ready for clinical use. J Allergy Clin Immunol.

[13] Virkud YV, Burks a W, Steele PH, Edwards LJ, Berglund JP, Jones SM (2017). Novel baseline predictors of adverse events during oral immunotherapy in children with peanut allergy. J Allergy Clin Immunol.

[14] Vazquez-Ortiz M, Alvaro-Lozano M, Alsina L, Garcia-Paba MB, Piquer-Gibert M, Giner-Munoz MT (2013). Safety and predictors of adverse events during oral immunotherapy for milk allergy: severity of reaction at oral challenge, specific IgE and prick test. Clin Exp Allergy.

[15] Rolinck-Werninghaus C, Staden U, Mehl A, Hamelmann E, Beyer K, Niggemann B (2005). Specific oral tolerance induction with food in children: transient or persistent effect on food allergy?. Allergy..

[16] Loh W, Tang M (2018). Adjuvant therapies in food immunotherapy. Immunol Allergy Clin N Am.

[17] Kobernick AK, Burks AW (2016). Active treatment for food allergy. Allergol Int.

[18] Prescott SL, Smith P, Tang M, Palmer DJ, Sinn J, Huntley SJ (2008). The importance of early complementary feeding in the development of oral tolerance: concerns and controversies. Pediatr Allergy Immunol.

[19] Tang MLK, Ponsonby AL, Orsini F, Tey D, Robinson M, Su EL (2015). Administration of a probiotic with peanut oral immunotherapy: a randomized trial. J Allergy Clin Immunol.

[20] Keet CA, Wood RA (2014). Emerging therapies for food allergy. J Clin Invest.

[21] De Kivit S, Saeland E, Kraneveld AD, Van De Kant HJG, Schouten B, Van Esch BCAM (2012). Galectin-9 induced by dietary synbiotics is involved in suppression of allergic symptoms in mice and humans. Allergy Eur J Allergy Clin Immunol..

[22] de Kivit S, Kostadinova AI, Kerperien J, Morgan ME, Muruzabal VA, Hofman GA (2017). Dietary, nondigestible oligosaccharides and *Bifidobacterium breve* M-16V suppress allergic inflammation in intestine via targeting dendritic cell maturation. J Leukoc Biol.

[23] Vonk MM, Diks MAP, Wagenaar L, Smit JJ, Pieters RHH, Garssen J, et al. Improved Efficacy of Oral Immunotherapy Using Non-Digestible Oligosaccharides in a Murine Cow’s Milk Allergy Model: A Potential Role for Foxp3+ Regulatory T Cells. Front Immunol. 2017;8. 10.3389/fimmu.2017.01230. 10.3389/fimmu.2017.01230 PMC5626810 29033945

[24] Hayen SM, Kostadinova AI, Garssen J, Otten HG, Willemsen LEM (2014). Novel immunotherapy approaches to food allergy. Curr Opin Allergy Clin Immunol.

[25] Wagenaar L, Bol-Schoenmakers M, Giustarini G, Vonk MM, van Esch BCAM, Knippels LMJ, et al. Dietary supplementation with nondigestible oligosaccharides reduces allergic symptoms and supports low dose Oral immunotherapy in a Peanut allergy mouse model. Mol Nutr Food Res. 2018:1800369. 10.1002/mnfr.201800369. 10.1002/mnfr.201800369 PMC6766954 30102006

[26] van Esch BC, van Bilsen JH, Jeurink PV, Garssen J, Penninks AH, Smit JJ (2013). Interlaboratory evaluation of a cow’s milk allergy mouse model to assess the allergenicity of hydrolysed cow’s milk based infant formulas. Toxicol Lett.

[27] Smit JJ, Willemsen K, Hassing I, Fiechter D, Storm G, van Bloois L (2011). Contribution of classic and alternative effector pathways in peanut-induced anaphylactic responses. PLoS One.

[28] Hartemink AJ. Principled computational methods for the validation and discovery of genetic regulatory networks. Massachusetts Inst Technol. 2001:1–206.

[29] Scutari M (2010). Learning Bayesian networks with the {bnlearn} {R} package. J Stat Softw.

[30] Hand DJ, Till RJ (2001). A simple generalisation of the area under the ROC curve for multiple class classification problems. Mach Learn.

[31] Brito MR, Chávez EL, Quiroz AJ, Yukich JE (1997). Connectivity of the mutual k-nearest-neighbor graph in clustering and outlier detection. Stat Probab Lett.

[32] Blondel VD, Guillaume J-L, Lambiotte R, Lefebvre E (2008). Fast unfolding of communities in large networks. J Stat Mech Theory Exp.

[33] Hamilton RG (2014). Allergic sensitization is a key risk factor for but not synonymous with allergic disease. J Allergy Clin Immunol.

[34] Ludman S, Shah N, Fox AT (2013). Managing cows’ milk allergy in children. BMJ..

[35] Al-Ahmed N, Alsowaidi S, Vadas P (2008). Peanut allergy: an overview. Allergy Asthma Clin Immunol.

[36] Vonk MM, Wagenaar L, Pieters RHH, Knippels LMJ, Willemsen LEM, Smit JJ (2017). The efficacy of oral and subcutaneous antigen-specific immunotherapy in murine cow’s milk- and peanut allergy models. Clin Transl Allergy.

[37] Moro G, Arslanoglu S, Stahl B, Jelinek J, Wahn U, Boehm G (2006). A mixture of prebiotic oligosaccharides reduces the incidence of atopic dermatitis during the first six months of age. Arch Dis Child.

[38] Hirata SI, Kunisawa J (2017). Gut microbiome, metabolome, and allergic diseases. Allergol Int.

[39] Tan J, McKenzie C, Vuillermin PJ, Goverse G, Vinuesa CG, Mebius RE (2016). Dietary Fiber and bacterial SCFA enhance Oral tolerance and protect against food allergy through diverse cellular pathways. Cell Rep.

[40] Trompette A, Gollwitzer ES, Yadava K, Sichelstiel AK, Sprenger N, Ngom-Bru C (2014). Gut microbiota metabolism of dietary fiber influences allergic airway disease and hematopoiesis. Nat Med.

[41] Canani RB, Sangwan N, Stefka AT, Nocerino R, Paparo L, Aitoro R (2016). Lactobacillus rhamnosus GG-supplemented formula expands butyrate-producing bacterial strains in food allergic infants. ISME J.

[42] Wang E, Zaman N, Mcgee S, Milanese JS, Masoudi-Nejad A, O’Connor-McCourt M (2015). Predictive genomics: a cancer hallmark network framework for predicting tumor clinical phenotypes using genome sequencing data. Semin Cancer Biol.

[43] McGee SR, Tibiche C, Trifiro M, Wang E (2017). Network analysis reveals a signaling regulatory loop in the PIK3CA-mutated breast Cancer predicting survival outcome. Genomics, Proteomics Bioinforma.

