# Supplementary figures and images for: A network-based approach for identifying suitable biomarkers for oral immunotherapy of food allergy
Source: BMC Bioinformatics. 2019 Apr 23;20:206. doi: 10.1186/s12859-019-2802-9 (PMC6480866; doi:10.1186/s12859-019-2802-9)

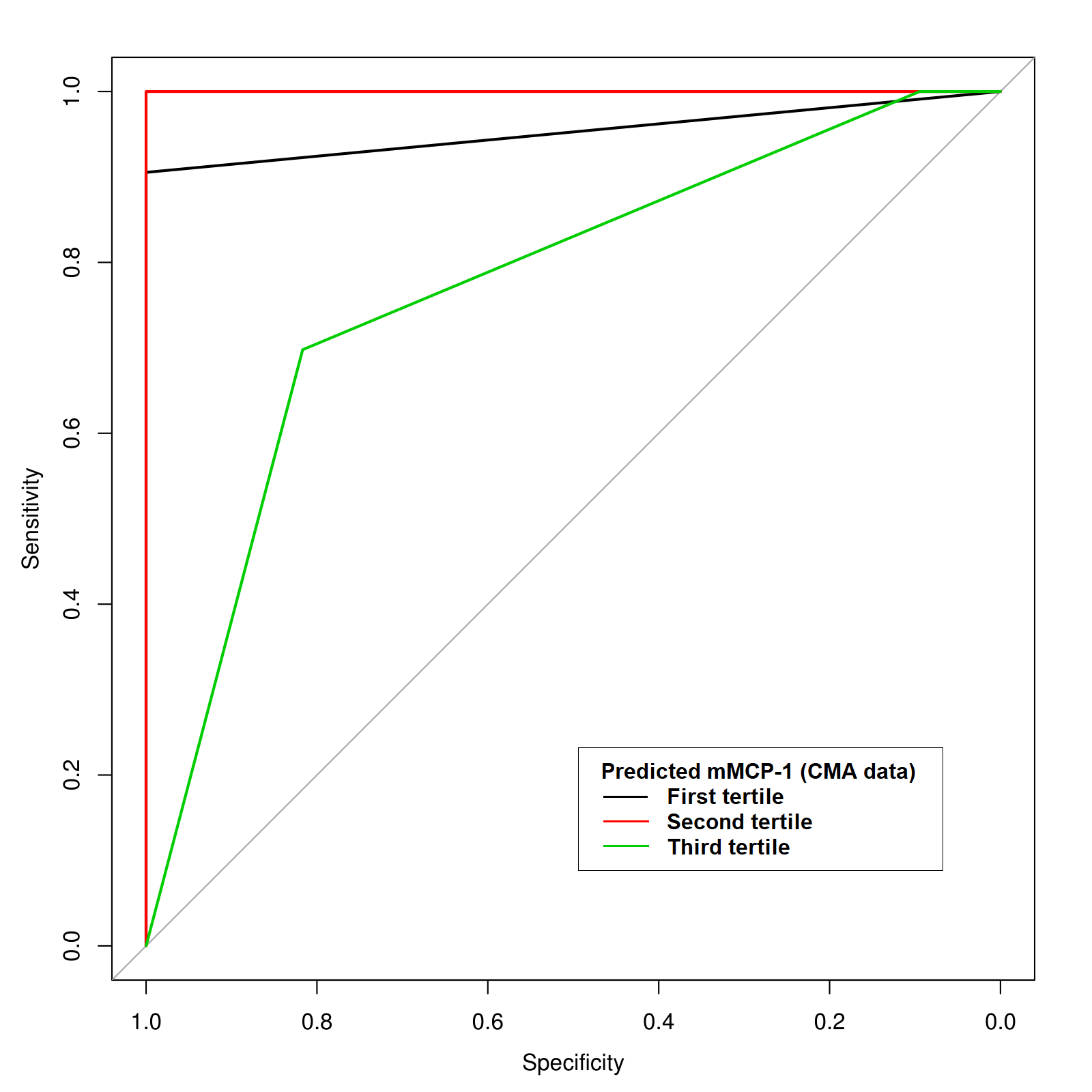

Supplement: Supplementary file 2 — Receiver operating characteristic (ROC) curves for the predicted mMCP-1 tertiles of the CMA data. Data were obtained from 91 animals. The curves correspond to a multiclass area under the curve value of 0.86, as calculated using the algorithm in [30]. (TIFF 365 kb) [file 12859_2019_2802_MOESM2_ESM.tiff]

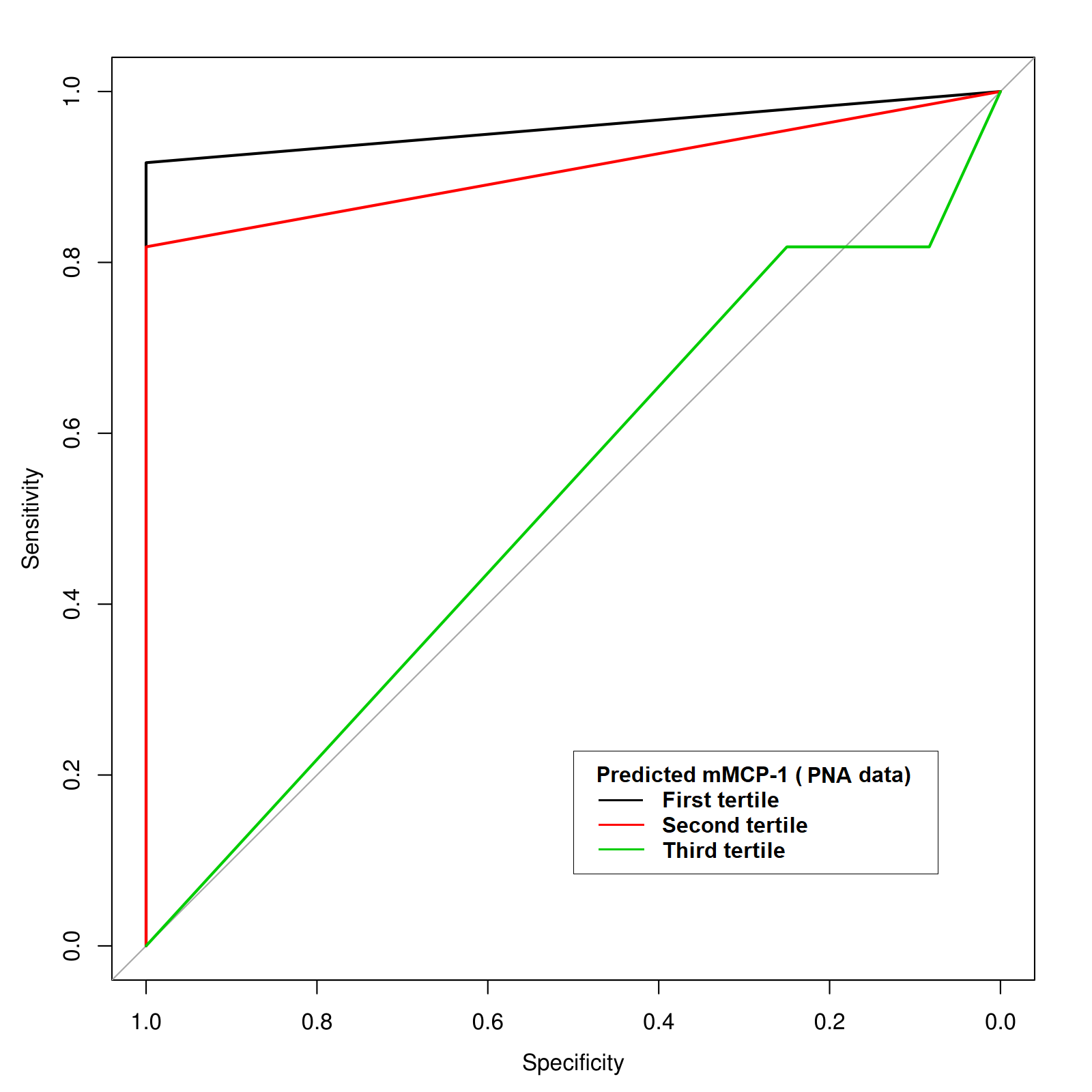

Supplement: Supplementary file 3 — Receiver operating characteristic (ROC) curves for the predicted mMCP-1 tertiles of the PNA data. Data were obtained from 67 animals. The curves correspond to a multiclass area under the curve value of 0.76, as calculated using the algorithm in [30]. (TIFF 370 kb) [file 12859_2019_2802_MOESM3_ESM.tiff]
